# PP06 Exploring Domains And Approaches In Medical Device Technology Evaluation: A Systematic Review

**DOI:** 10.1017/S0266462325102262

**Published:** 2025-12-29

**Authors:** Leidy Anne Teixeira, Fotini Toscas, Daiana Blas, Vania Cristina Canuto Santos

## Abstract

**Introduction:**

Medical device technology evaluation has advanced significantly in recent years. This study was conducted with the aim of identifying the main domains used globally for the evaluation of these technologies and considering the perspective of technology adoption in healthcare systems.

**Methods:**

Structured searches were conducted in MEDLINE, Embase, the Virtual Health Library, the Cochrane Library, and Web of Science databases using the terms “medical technology” OR “medical device” OR “equipment and supplies” AND “health technology assessment” OR “biomedical technology assessments” for studies published between January 2017 and May 2023, excluding those on specific technologies or audiences. Documents from the International Network of Agencies for Health Technology Assessment and databases from the World Health Organization were also included. Study selection, extraction, and quality assessment were performed by two independent reviewers, with discrepancies resolved by a third reviewer. The term “domain” in this systematic review was inspired by the EUnetHTA Core Model.

**Results:**

A total of 5,790 studies were retrieved, with 41 meeting the inclusion criteria. Findings were grouped into eight domains, with percentages based on the selected articles (n=41; 100%): (i) safety (n=17; 41%); (ii) efficacy (n=18; 44%); (iii) health problem, current use of technology, and innovation (n=17; 41%); (iv) legal aspects (n=28; 68%); (iv) organizational benefits (n=17; 41%); (vi) description and technical characteristics of the technology (n=30; 73%); (vii) costs and economic evaluation (n=24; 58%); and (viii) social participation (n=11; 27%). Most articles (80%) were of European origin.

**Conclusions:**

Due to the diversity of medical devices, a single methodological guideline may not cover all their specificities. Studies suggested grouping by technological characteristics to standardize the process. We highlight the importance of exploring less discussed domains, such as social participation and organizational benefits, which represent future topics.

